# Supplementary material for: MOST: a modified MLST typing tool based on short read sequencing
Source: PeerJ. 2016 Aug 17;4:e2308. doi: 10.7717/peerj.2308 (PMC4991843; doi:10.7717/peerj.2308)
Supplement: Supplemental Information 1 [file peerj-04-2308-s001.doc]

**Supplementary methods**

The following amendments were made to SRST (version 1):

(1) In the SRST (version1) software, blast is used to determine the flanking regions. Occasionally SRST (version 1) determines incorrect allele flanking regions because the blast hit does not exactly match the start of the query 'bait' sequence. The code was now amended to extract the correct flanking region.

(2) SRST (version 1) uses BWA global alignment. BWA global alignment was replaced by Bowtie2 global alignment because Bowtie2 (with very sensitive options) is more sensitive than BWA. Bowtie2 reports secondary alignments directly in the Sam file and sets a secondary alignment bit (256) in the flag. However, reads with 256 bit set were not reported in the samtools pileup. To solve this 256 was removed from the flag value in the Sam file.

(3) Anomalous read pairs were counted using the -A flag when using samtools variant calling software

(4) INDELs were reported to discriminate alleles that are different due to insertion and deletion.

(5) The code was amended in order to calculate the average consensus depth values correctly.

(6) Bowtie2/2.2.5 occasionally generates an I0 error. This was caused by two temporary files remaining in the /tmp folder location of the file system. These files were named <processnumber>.inpipe1 and <processnumber>.inpipe2. The errors occur when a large number of samples are processed in parallel on the system, and the same process number is reused. To rectify this the Bowtie2 Perl script was changed in such a way that these files are written to a temporary folder within the /tmp location.

The folder is then removed on program exit. The changes are:

added

Line 1: use File::Temp qw(tempdir);

Line 351: $temp_dir = tempdir(CLEANUP=>1;
